# The pleiotropic effects of prebiotic galacto-oligosaccharides on the aging gut

**DOI:** 10.1186/s40168-020-00980-0

**Published:** 2021-01-28

**Authors:** Jason W. Arnold, Jeffery Roach, Salvador Fabela, Emily Moorfield, Shengli Ding, Eric Blue, Suzanne Dagher, Scott Magness, Rita Tamayo, Jose M. Bruno-Barcena, M. Andrea Azcarate-Peril

**Affiliations:** 1 Department of Medicine, Division of Gastroenterology and Hepatology, School of Medicine, University of North Carolina, Chapel Hill, NC USA; 2 UNC Microbiome Core, Center for Gastrointestinal Biology and Disease (CGIBD), School of Medicine, University of North Carolina, Chapel Hill, NC USA; 3 UNC Information Technology Services and Research Computing, University of North Carolina, Chapel Hill, NC USA; 4 Current affiliation: Programa de Inmunología Molecular Microbiana. Departamento de Microbiología y Parasitología, Facultad de Medicina, Universidad Nacional Autónoma de Mexico, Mexico City, Mexico; 5 Department of Cell Biology and Physiology, University of North Carolina, Chapel Hill, NC USA; 6 Department of Plant and Microbial Biology, North Carolina State University, Raleigh, NC USA; 7 Joint Department of Biomedical Engineering, University of North Carolina, Chapel Hill and North Carolina State University, Raleigh, NC USA; 8 Department of Microbiology and Immunology, University of North Carolina, Chapel Hill, NC USA

**Keywords:** Gut microbiome, Prebiotics, *Bifidobacterium*, Intestinal permeability, Host-microbiota interactions, Diet, Antibiotics, Metagenomics, Transcriptomics, Organoids

## Abstract

**Background:**

Prebiotic galacto-oligosaccharides (GOS) have an extensively demonstrated beneficial impact on intestinal health. In this study, we determined the impact of GOS diets on hallmarks of gut aging: microbiome dysbiosis, inflammation, and intestinal barrier defects (“leaky gut”). We also evaluated if short-term GOS feeding influenced how the aging gut responded to antibiotic challenges in a mouse model of *Clostridioides difficile* infection. Finally, we assessed if colonic organoids could reproduce the GOS responder—non-responder phenotypes observed in vivo.

**Results:**

Old animals had a distinct microbiome characterized by increased ratios of non-saccharolytic versus saccharolytic bacteria and, correspondingly, a lower abundance of β-galactosidases compared to young animals. GOS reduced the overall diversity, increased the abundance of specific saccharolytic bacteria (species of *Bacteroides* and *Lactobacillus*), increased the abundance of β-galactosidases in young and old animals, and increased the non-saccharolytic organisms; however, a robust, homogeneous bifidogenic effect was not observed. GOS reduced age-associated increased intestinal permeability and increased *MUC2* expression and mucus thickness in old mice. Clyndamicin reduced the abundance *Bifidobacterium* while increasing *Akkermansia*, *Clostridium*, *Coprococcus*, *Bacillus*, *Bacteroides*, and *Ruminococcus* in old mice. The antibiotics were more impactful than GOS on modulating serum markers of inflammation. Higher serum levels of IL-17 and IL-6 were observed in control and GOS diets in the antibiotic groups, and within those groups, levels of IL-6 were higher in the GOS groups, regardless of age, and higher in the old compared to young animals in the control diet groups. RTqPCR revealed significantly increased gene expression of TNFα in distal colon tissue of old mice, which was decreased by the GOS diet. Colon transcriptomics analysis of mice fed GOS showed increased expression of genes involved in small-molecule metabolic processes and specifically the respirasome in old animals, which could indicate an increased oxidative metabolism and energetic efficiency. In young mice, GOS induced the expression of binding-related genes. The galectin gene *Lgals1*, a β-galactosyl-binding lectin that bridges molecules by their sugar moieties and is an important modulator of the immune response, and the PI3K-Akt and ECM-receptor interaction pathways were also induced in young mice. Stools from mice exhibiting variable bifidogenic response to GOS injected into colon organoids in the presence of prebiotics reproduced the response and non-response phenotypes observed in vivo suggesting that the composition and functionality of the microbiota are the main contributors to the phenotype.

**Conclusions:**

Dietary GOS modulated homeostasis of the aging gut by promoting changes in microbiome composition and host gene expression, which was translated into decreased intestinal permeability and increased mucus production. Age was a determining factor on how prebiotics impacted the microbiome and expression of intestinal epithelial cells, especially apparent from the induction of galectin-1 in young but not old mice.

**Video abstract**

**Supplementary Information:**

The online version contains supplementary material available at 10.1186/s40168-020-00980-0.

## Background

The fragility of the gut microbiota and consequent susceptibility to disease are accentuated at the beginning and at the end of life. The aging gut microbiome has a demonstrated altered bacterial diversity with reductions in the abundance of beneficial microorganisms [1–12]. Imbalances in the gut microbiota promote a basal inflammatory state and enhance susceptibility to viral and bacterial infections, including *Clostridioides difficile* [13–15]. The elderly human gut microbiome has been reported to have a reduced abundance of *Bifidobacterium*, *Faecalibacterium prausnitzii*, and *Clostridium* XIVa [2–4] and increased *Clostridium perfringens*, coliforms, enterococci [1], *Streptococcus*, *Staphylococcus*, and *Enterobacteria* [2, 3, 5–7]. Accordingly, the aging human gut microbiota shows a loss of genes involved in the production of short-chain fatty acids (SCFAs) and a decrease in saccharolytic potential, with a reduced representation of starch, sucrose, galactose, glycolysis, and gluconeogenesis metabolism pathways; a concomitant loss of fibrolytic microorganisms; and an overall increase in proteolytic function [16]. Consistent with human microbiome observations, old mice have a distinctive gut microbiome characterized by lower phylogenetic diversity, increased representation of potentially pathogenic taxa including *Rikenella* and *Enterobacteriaceae*, and reduced representation of di-, oligo-, and polysaccharide utilization genes [17].

The ability of bacteria to access host tissues is limited by the mucus layer in healthy individuals. In mice, the colon of old animals has a thinner firm mucus layer (< 10 μm) compared to young mice (20–25 μm), resulting in a failure to spatially compartmentalize the microbiota to the intestinal lumen [18, 19]. However, the number of mucus-producing goblet cells does not decline in the specialized follicle-associated epithelium in aged mice [20]. The mucus protein composition is relatively homogeneous along the intestine; however, the main mucin component synthesized and secreted by intestinal goblet cells, MUC2, shows region-specific *O*-glycan patterns [21, 22]. Changes in properties of the mucus barrier have been associated with shifts in bacterial community composition [23]. Conversely, beneficial microorganisms like *Lactobacillus plantarum* [24] and *Akkermansia muciniphila* [25] have been shown to promote an increase in mucus thickness and improve host tight junctions in aging animals, reducing permeability. Likewise, *Bifidobacterium longum* and *B. longum* subsp*. infantis* provided protection against deterioration of the colonic mucus layer, counteracting negative influences of Western diets on mucus hyper-degradation by enhancing production [26, 27].

An increased inflammatory state is another hallmark of gut aging [28]. Aging changes the balance between inflammatory and anti-inflammatory cytokines favoring an excessive production of IL-6, TNFα, and IL-1β, directly affecting intestinal permeability [28, 29]. Traditionally, immuno-senescence, which is a decrease in the efficiency of the immune system over time, has been considered the largest contributor to increases in inflammatory mediators [30, 31]. However, recent studies have highlighted a prominent role of dysbiotic states of the gut microbiota in inflammatory bowel conditions and metabolic diseases [32, 33], which show age-related increases in incidence [34].

Prebiotic β(1-4) galacto-oligosaccharides (GOS) are complex carbohydrates that are resistant to digestion in the upper gastrointestinal tract. GOS arrive at the colon intact and consequently increase the abundance of specific primary and secondary degraders, resulting in an expanded probiome (beneficial members of the intestinal microbiota) [35]. GOS and fructo-oligosaccharides are the preferred prebiotics currently added to commercial infant formula to mimic the beneficial effects of the human milk oligosaccharides (HMOs) in breast milk [36]. Akkerman et al. [37] recently reviewed the effects of non-digestible carbohydrates in infant formulas as substituents of HMOs on the gut microbiota and maturation and stated that, beyond a well-established role in bifidogenesis, GOS also act as soluble decoy receptors to prevent adhesion of pathogens to epithelial cells, stimulate tight junctions, enhance intestinal barrier function through modulation of goblet cells [38], and reduce the release of the inflammatory marker CXCL8 by Caco-2 cells [39]. In addition, GOS support intestinal development in piglets, increasing the expression levels of β-defensins-2 and sIgA, suggesting improvement of mucosal immune responses [40]. In adults, increases in the abundance of *Bifidobacterium* upon GOS consumption have been reported in humans and animal models [41–45]. We demonstrated that specific bifidobacteria (*B. longum*, *B. adolescentis*, *B. catenulatum*, and *B. breve*) increased when lactose-intolerant adults received purified GOS [46, 47]. We also demonstrated that pure GOS are capable of increasing the abundance of beneficial bacteria including *Faecalibacterium prausnitzii* and species of *Lactobacillus*, *Christensenellaceae*, *Collinsella*, *Prevotella*, and *Catenibacterium* [45, 47]*.* In addition, GOS directly induce the expression of *MUC2*, *TFF3*, and *RETNLB* in the colonic adenocarcinoma LS174T cell line, which exhibits a goblet cell-like phenotype [38]. Finally, studies in humans showed that GOS significantly increased the numbers of bifidobacteria, phagocytosis, NK cell activity, and anti-inflammatory IL-10 in healthy elderly individuals, with a significant reduction in the production of pro-inflammatory cytokines (IL-6, IL-1β, and tumor necrosis factor-α) [48].

Based on the demonstrated effects of GOS on infants and adults, our study aimed to determine the impact of pure GOS [49] on the hallmarks of gut aging. We also evaluated the effect of short-term GOS feeding on how the aging gut microbiome responds to antibiotic challenges, since these interventions are common and relevant in older adults. In fact, in the years 2007 to 2009, patients aged ≥ 65 years used more antimicrobials, at 1.10 per person per year, compared to 0.88 antimicrobials used per person per year in patients aged 0–64 years [50]. Antibiotics induce gut microbiome disturbances, persistent through the constant presence in the food supply [51] or by introducing new and potentially stable changes with each cycle of antibiotic administration [52, 53]. Amidst these microbiota disturbances, the prevalence of infection by opportunistic pathogens, including *Clostridioides difficile*, is dramatically overrepresented in elderly populations [13]; thus, we evaluated if short-term GOS feeding influenced how the aging gut responded to the antibiotic challenges of a model of *Clostridioides difficile* infection to lay down the groundwork for future studies focused on prebiotics as preventive treatments against infection. Finally, we evaluated if colonic organoids [54] reproduced the in vivo response to GOS. Our findings add further evidence to previous limited studies on age-associated dysbiosis and intestinal physiology, providing valuable insights into how dietary GOS impact the microbiome composition and functionality, intestinal barrier function, and biomarkers of inflammation in an animal model of aging.

## Results

### Impact of GOS and antibiotics on the gut microbiome

**Fig. 1:**
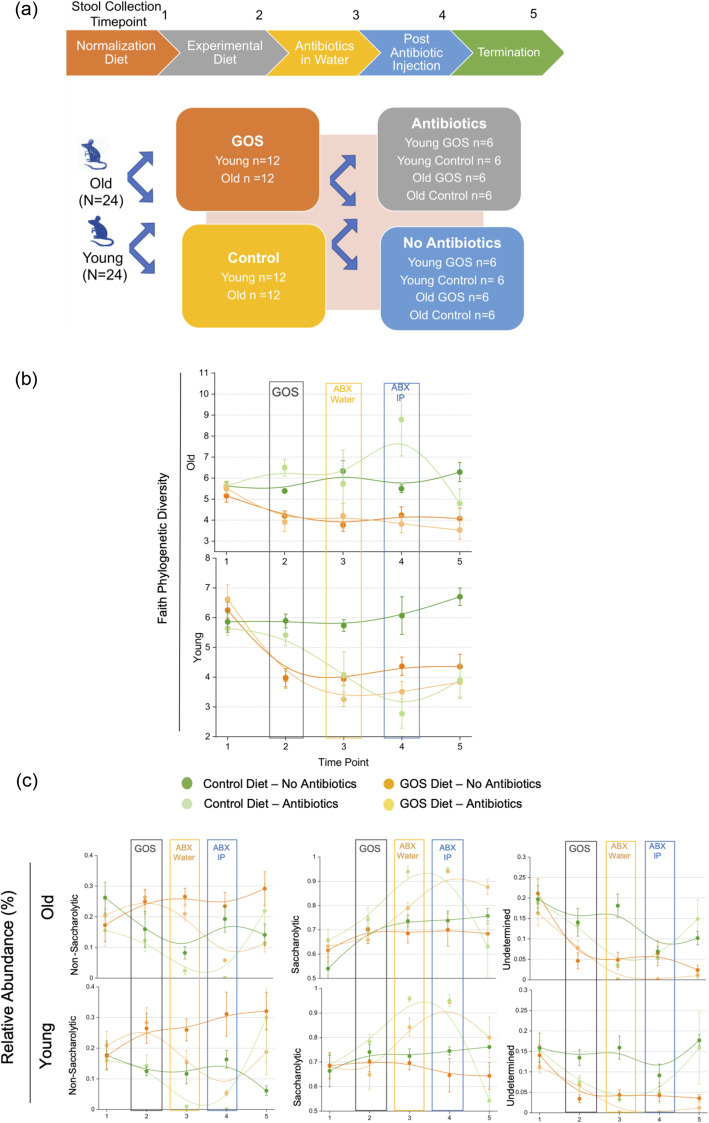
Prebiotic GOS and antibiotics decrease microbiome diversity in the gut. (**a**). Experimental design outlining time points T1 (post-standardization), T2 (post-specialized diet), T3 (post-antibiotics in water), T4 (post-clindamycin IP injection), and T5 (pre-sacrifice) (*N* = 48) (**b**). Impact of the different treatments on phylogenetic diversity. GOS reduced diversity in both old and young animals. Alpha diversity declined with GOS consumption (T2) and antibiotic administration (T3, in young mice) and remained consistent through T5. **c** Relative abundance of saccharolytic, non-saccharolytic, and bacteria of undetermined metabolism over time. Line colors are as in Fig. 1b

A cohort of twenty-four young (6 weeks) and old (60 weeks) female C57BL/6J SPF mice (*N* = 48) were fed the control diet for 20 days (microbiome normalization period) and then switched to the experimental diet containing prebiotics for an additional 2 weeks (Fig. 1a). Analysis of 16S rRNA amplicon sequencing data performed on longitudinal time points 1 (T1, day 20, after normalization period), 2 (T2, day 35), 3 (T3, day 38), 4 (T4, day 42), and 5 (T5, day 50) assigned 190 distinct bacterial taxa, when the analysis was performed at the equivalent of species-level in QIIME2.

Analysis of alpha diversity revealed a decrease in phylogenetic diversity in old and young animals receiving the prebiotics diet at T2, day 35 (repeated measures ANOVA with pairwise comparisons *P* < 0.05) (Fig. 1b). Introduction of antibiotics at T3 reduced diversity in control-fed young and had a variable effect in old animals but had little impact on GOS-fed old mice. These antibiotic-driven changes in alpha diversity persisted through T4 and T5, revealing higher diversity in antibiotic-free, control-fed animals in both young and old animals. Animals did not lose weight during the antibiotic treatments. Likewise, none of the animals exhibited any signs of gastrointestinal distress or reduced activity before the clindamycin IP injection. After administration of clindamycin, mice exhibited temporary runny stools (T4).

Analysis of saccharolytic and non-saccharolytic bacteria (Fig. 1c) showed that GOS increased the relative abundance of mostly non-saccharolytic bacteria, driven by *Akkermansia*, (Figure S1), reducing the abundance of bacteria of undetermined metabolism at T2. These changes persisted through T5 in animals that were not treated with antibiotics, but additional shifts were observed in antibiotic-treated animals, increasing the abundance of saccharolytic taxa at T3–T4 with reductions in both non-saccharolytic and undetermined taxa. Although the bifidogenic effect of GOS has been previously reported by us and others [41, 45, 47, 55], we did not observe a consistently significant increase in the abundance of bifidobacteria in old or young animals fed GOS diet, also in accordance with previous reports of the responder-non-responder phenotype (Fig. 6a, left).

**Fig. 2:**
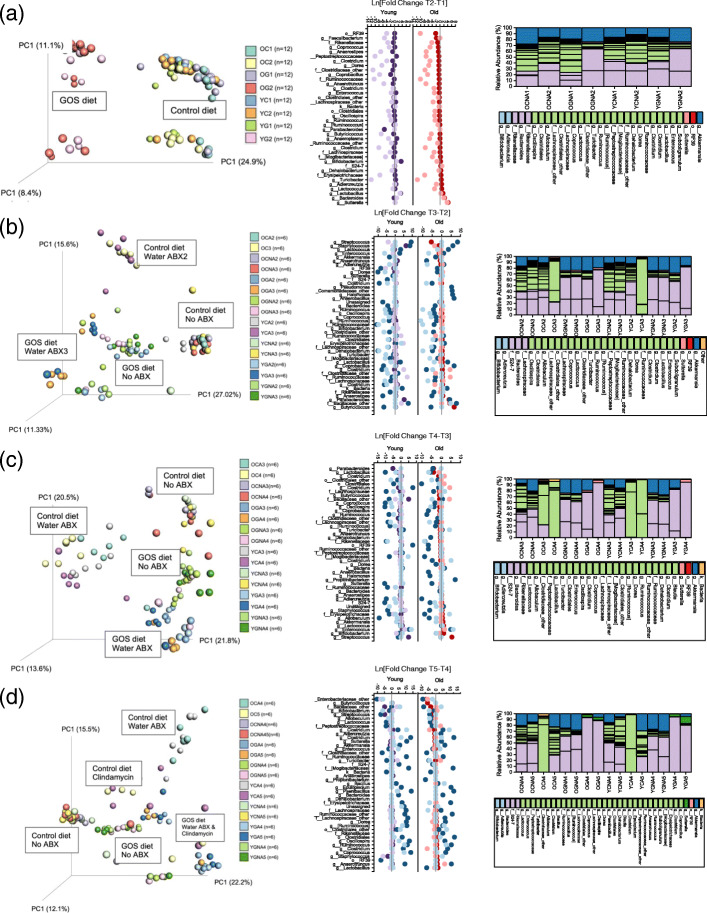
Unweighted UniFrac PCoA plots and differences in the relative abundance of specific bacteria at different time points and treatments (**a**). The T1–T2 PCoA plot (left) revealed the baseline differences between old and young mice (OC, old mice, control diet; OG, old mice, GOS diet; YC, young mice, control diet; YG, young mice, GOS diet). The middle panel shows the prebiotic impact on young (light purple) and old (pink) mice compared to the control diet (purple = young control, red = old control). The right panel shows the composed differences in relative abundances by age, diet, and time point. **b** The T2–T3 PCoA plot (left) and genus-level analysis (middle) showed the impact of the antibiotic treatment in water on both diet groups. Middle panel: control and GOS diets as before. Light blue dots represent no antibiotic treatment, and dark blue dots represent antibiotic treatment. **c** The T3–T4 PCoA plot (left) and genus-level analysis (middle) represent the samples immediately after clindamycin IP injection. The graphs did not show dramatic impacts to the microbiome, which were clearly visible in **d** the T4–T5 PCoA and taxa plots

Principal coordinate analysis (PCoA) plots showed significant differences in microbiome composition between old and young mice prior to GOS treatment (ANOSIM *R* = 0.45, *P* = 0.001, PERMANOVA pseudo-*F* = 3.8, *P* = 0.001) at day 20 (T1) (Fig. 2a). We observed clustering of GOS-fed old and young mice (OG2 and YG2) away from control-fed animals and sub-clusters at T1 that separated control old and young animals. The separation between GOS and control clusters persisted in T2–T3, and sub-clustering was determined by the first antibiotic treatment. A more extreme effect of the antibiotics was observed in mice fed the control diet at T4 (Fig. 2c). At this time, the separation by diet persisted, but the clustering by age in the non-antibiotic control young and old mice was not observed. Finally, we observed clustering by diet and antibiotics at T5, with samples from both antibiotic treatments grouping together in the GOS animals but not control. These results indicate a clear influence of diet on the response of the microbial community to antibiotics. Analysis at the genus level identified 48 taxa significantly changed from T4 to T5 upon clindamycin injection in at least one of the groups in our analysis (Old_control, Old_GOS, Young_control, Young_GOS) (Supplementary Text). Interestingly, while the antibiotic decreased the abundance of *Bifidobacterium* and *Lactobacillus* in young and old mice in both diets, clindamycin increased the relative abundance of non-saccharolytic bacteria in animals (young and old) receiving the control diet.

**Fig. 3:**
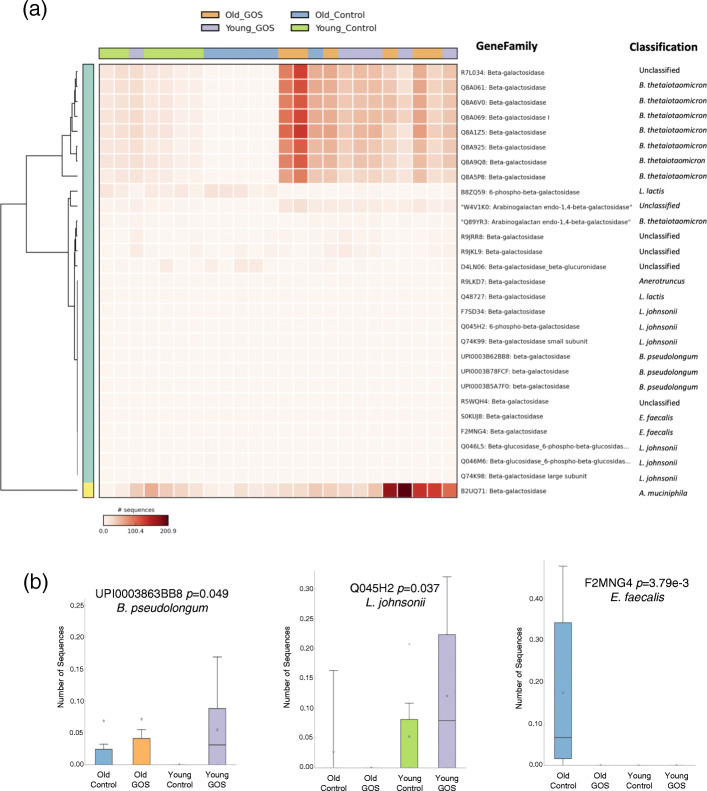
**a** Whole-genome shotgun (WGS) sequencing of stool samples showed that GOS treatment significantly modified the abundance of 22 out of 29 of beta-galactosidases in old and young mice (*Kruskal-Wallis FDR-corrected *P* < 0.05). **b** Representative patterns of abundance of beta-galactosidases from *B. pseudolongum*, *L. johnsonii*, and *E. faecalis*

To investigate whether compositional differences were translated into potential functional discrepancies, we conducted whole-genome shotgun (WGS) sequencing of stool collected from 6 old and 6 young animals (not treated with antibiotics) at time point 1 (control diet) and at time point 5 (GOS diet). First degraders encoding β-galactosidases and β-glucosidases metabolize GOS in the colon generating lactate and acetate [56]. In the healthy adult gut, secondary degraders including *Faecalibacterium* [57] and *Roseburia* [58] utilize these primary fermentation products to generate butyrate, which directly benefits host physiology [59–61]. The gene family output of HUMAnN2 identified 29 entries as β-galactosidases, the essential enzymes for the initial catabolism of GOS by primary degraders. Those included β-galactosidases (EC:3.2.1.23), phospho-β-galactosidases (EC:3.2.1.85), and arabinogalactan endo-1,4- β-galactosidases (EC: 3.2.1.89). β-galactosidases catalyze the hydrolysis of terminal, non-reducing beta-d-galactoside residues, while phospho-β galactosidases hydrolyze 6-phospho- β-d-galactoside residues. Arabinogalactan endo-1,4- β-galactosidases hydrolyze (1➔4)-β-d-galactosidic linkages in type I arabinogalactans. Clustering of relative abundance of β-galactosidases showed that most samples in the old control group had significantly (Kruskal-Wallis FDR corrected *P* < 0.05) reduced abundance of unclassified β-galactosidases as well as β-galactosidases from *Bacteroides thetaiotaomicron* and *Akkermansia muciniphila*, abundance of which was increased in the old GOS group (Fig. 3a). Interestingly, most samples from control young and control old clustered within their own group, while GOS-fed mice (young and old) clustered mostly in one group. Figure 3b shows the box plots of representatives from *Bifidobacterium pseudolongum* (increased by GOS in both young and old animals), *Lactobacillus johnsonii* (only increased by GOS in young animals), and *Enterococcus faecalis* (overrepresented in the old control group) that had low read counts, and hence, their pattern of representation could not be clearly assessed from the heatmap.

### GOS impact on intestinal permeability, biomarkers of inflammation, and mucin production

**Fig. 4:**
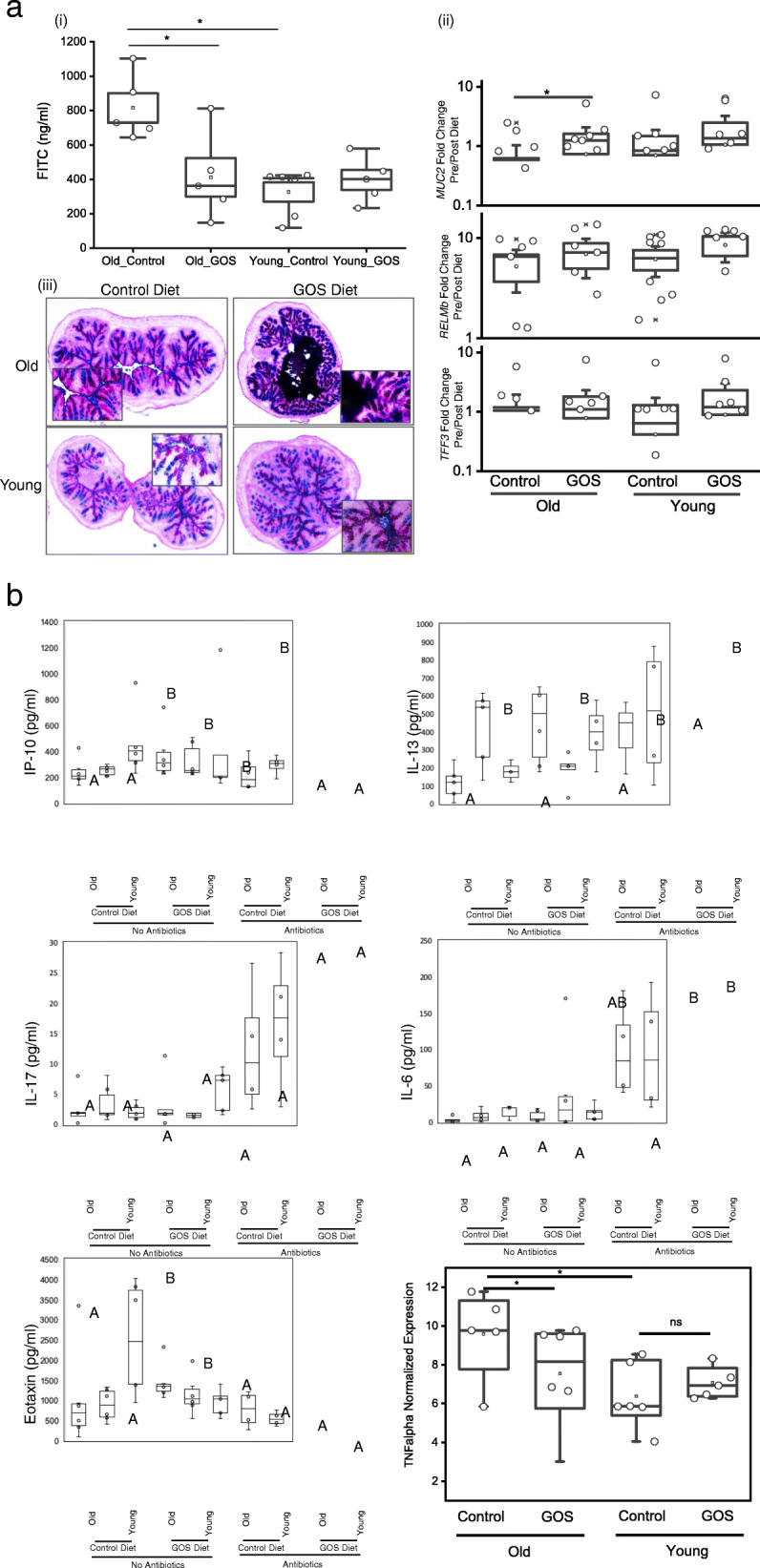
**a** (i) Old mice had higher intestinal permeability measured by FITC-dextran assays than young animals. (ii) Old mice fed GOS had significantly increased *MUC2* expression (*p < 0.05). The expression of *TFF3* and *RELMb* tended to increase in the GOS groups, but differences were not statistically significant. (iii) Paraformaldehyde vapor fixation and subsequent PAS staining showed increased mucus thickness in old mice fed the prebiotics diet. **b** Inflammatory biomarkers were modulated by antibiotics and GOS. A 2 × 2 × 2 ANOVA test showed (i) increased serum IP-10 in GOS-fed animals without antibiotic treatment and in antibiotic-treated animals fed control diet. (ii) Serum IL-13 levels were higher in young animals than in old animals in all groups. (iii) IL-17 levels were higher in antibiotic-treated animals than in animals without antibiotics. (iv) IL-6 was increased in antibiotic-treated old animals (GOS and control) compared to old animals without antibiotic treatments and elevated in young animals treated with both GOS and antibiotics. (v) Eotaxin levels were higher in GOS-fed animals without antibiotic treatment, but lower in GOS-fed animals that received antibiotics, regardless of age. (vi) Expression of TNFα quantified by RT-qPCR was higher in old animals compared to young and reduced by GOS treatment in old animals

Assessment of gut barrier function showed significantly increased intestinal permeability in old compared to young mice, with decreased values in old animals fed GOS (Fig. 4a(i)). No significant differences were observed between control and GOS diets in young animals. GOS diet increased the mucus abundance and thickness in the lumen of old mice (2 × 2 ANOVA *P* < 005) (Fig. 4a(iii)), but a less pronounced effect was observed in young animals. Imaging analysis of PAS-stained colon tissues using the ImageJ software confirmed the increased abundance of mucin/mucin-producing cells (blue/purple) in the old GOS group compared to all of the other groups (Figure S2).

Epithelial surface integrity is maintained in part by genes in the trefoil factor (TFF) family including TFF3 [62]. TFF3 binds to the cysteine-rich amino terminal von Willebrand factor (vWF) of MUC2 enhancing the protection of the gastrointestinal mucosa against injury through interactions with mucins [63]. The resistin-like molecule (RELM) family of proteins facilitate the formation of unique disulfide-dependent multimeric assembly units. RELMb is predominantly expressed by goblet cells and epithelial cells in the colon and is involved in the maintenance of colonic epithelial barrier function by upregulating *MUC2* [64]. The expression of *MUC2* was increased with GOS, while *RELMb* and *TFF3* showed non-significant increases (Fig. 4a(ii)).

Increased levels of circulating cytokines (plasma and serum) associated with aging have been reported in humans and mice [28, 65, 66]. In our study, the most important factor in modulating levels of serum cytokines was antibiotic administration (2 × 2 × 2 ANOVA *P* < 0.05). Overall, the antibiotics groups had higher IL-17 and IL-6 levels in both control and GOS diets. Within the antibiotics groups, the levels of IL-6 were higher in the GOS diet groups, regardless of age, and higher in the old compared to young animals in the control diet groups (Fig. 4b(iii and iv)). Antibiotic administration resulted in higher IP-10 and eotaxin in the control diet groups and lower IP-10 and eotaxin in the GOS diet groups (Fig. 4b(i and v)). Young animals had higher levels of IL-13, regardless of diet and antibiotics (Fig. 4b(ii)). Finally, although the levels of serum tumor necrosis factor (TNF)α did not show significant differences between the groups (not shown), RTqPCR analysis revealed significantly increased gene expression of TNFα in distal colon tissue of old mice, and prebiotics decreased the expression of the cytokine (Fig. 4b(vi)).

### Colon mucosa transcriptome profiling showed significant differences in response to GOS diet between young and old mice

**Fig. 5:**
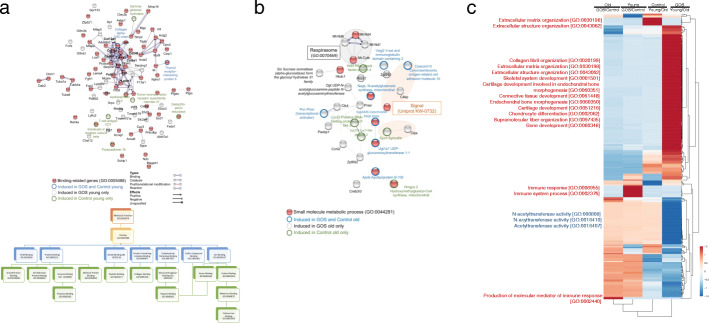
STRING network analysis [67] of expression data from colon showed different GOS effects on the intestinal epithelium of old and young mice. **a** GOS induced expression of binding-related genes (GO:0005488) in young mice while inducing **b** small-molecule metabolic processes genes (GO:0044281) in old animals. Network nodes represent predicted proteins. Splice isoforms or post-translational modifications are collapsed so each node represents all the proteins produced by a single, protein-coding gene locus. The confidence cutoff for showing interaction links was 0.900 (highest). The lower panel in **a** shows the most represented GO categories within binding-related genes in our transcriptomics data. **c** A heatmap of expression data revealed that GOS act as a modulator of the immune system in old and young mice. The heatmap was generated using ClustVis [68]. Rows were centered; unit variance scaling was applied to rows. Rows were clustered using correlation distance and average linkage

We next aimed to determine whether GOS induced similar changes in colon gene expression in young compared to old animals. Covariate analysis of transcriptomics data from colon tissue of 6- versus 60-week-old mice fed the control or GOS diets showed clear patterns of gene expression in pairwise group comparisons (Fig. 5, Supplementary Table 1). Old animals fed GOS had an increased representation of genes involved in the production of molecular mediators of the immune response (GO:0002440) compared to old animals fed the control diet. Functions included immunoglobulin production, positive regulation of T-helper 1 cell cytokine production, and positive regulation of immunoglobulin biosynthetic processes. Conversely, old animals fed GOS had a lower expression of genes in the collagen-containing extracellular matrix (GO:0062023), specifically a disintegrin-like and metallopeptidase (reprolysin type) with thrombospondin type 1 motif, collagen, type VI, alpha 1, fibronectin 1, and insulin-like growth factor binding protein 6 (Fig. 5a).

The majority of genes (92 genes) overexpressed in young compared to old animals fed the prebiotics diet were functionally associated with binding (GO:0005488), specifically protein binding (GO:0005515, 64 genes), ion binding (GO:0043167, 53 genes), and protein-containing complex binding (GO:0044877, 20 genes) (Fig. 5b). In old animals fed GOS, genes involved in small molecule metabolic processes (GO:0044281) and specifically the respirasome (GO:0070469) were overexpressed compared to young animals on the same diet (Fig. 5). In young mice, GOS also stimulated the expression of the galectin gene *Lgals1*, which encodes a β-galactosyl-binding lectin that bridges molecules by their sugar moieties, forming a signaling and adhesion network [69]. Galectins bind specifically to β-galactoside sugars and have been linked to host-microbe interactions by direct binding to microorganisms affecting their survival or function and modulating the innate or adaptive responses of immune cells against microbes either via extra- or intracellular mode of action. In fact, galectins can have direct antimicrobial effects [70]. Accordingly, further analysis of gene expression differences by mapping onto the Kyoto Encyclopedia of Genes and Genomes (KEGG) metabolic maps (www.genome.jp/kegg/) showed higher expression levels of genes in focal adhesion, PI3K-Akt, and ECM-receptor interaction pathways [71] in young compared to old animals. The lowest expressed gene in both old and young animals fed GOS was *Trpv6* (transient receptor potential cation channel, subfamily V, member 6), a highly selective calcium channel that acts via calcium absorption in the intestine and kidney [72]. This gene was overexpressed in young compared to old animals both on the GOS and control diets.

### Responders versus non-responders to GOS diets

**Fig. 6:**
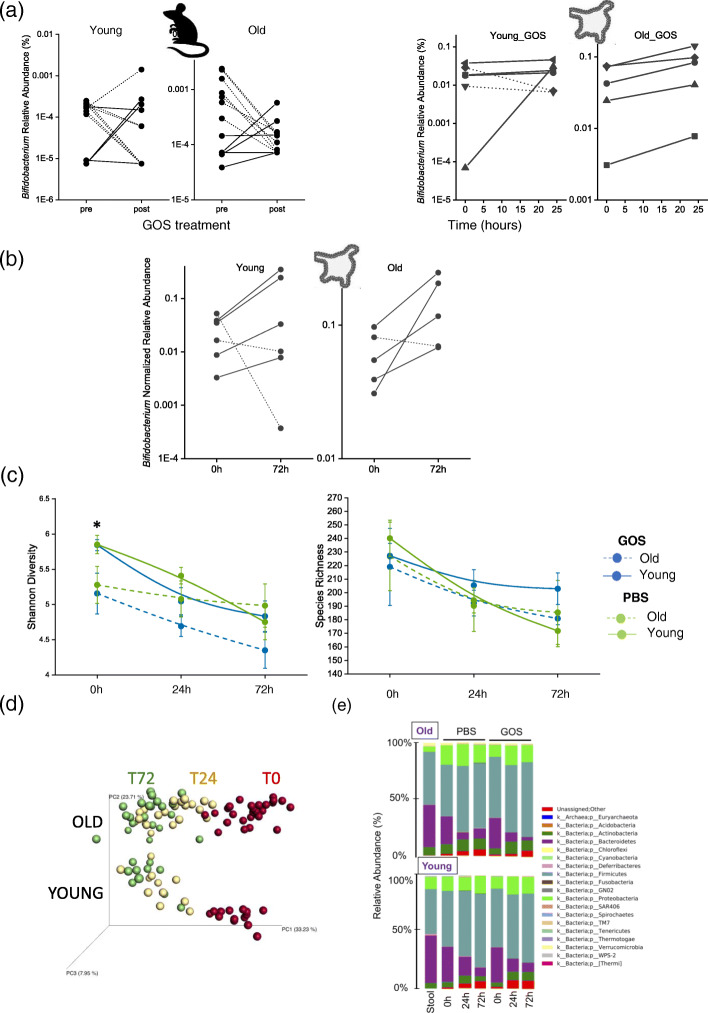
The variable bifidogenic effect observed in vivo was reproduced in vitro in the organoid platform as shown by **a** 16S rRNA amplicon sequencing and **b** high-throughput qPCR. **c** Shannon diversity and species richness within microbiota-colonized organoids declined over time. **d** Unweighted UniFrac PCoA plots revealed differences between old and young microbiota upon colonization in organoids, which converged to a single grouping over 72 h. **e** Taxonomy plots of microbiota-colonized organoids showed changes over time in communities derived from both old and young animals

It has been previously reported that, when fed GOS, a proportion of individuals will not mount a bifidogenic response [41, 45, 47], i.e., they will not display an appreciable increase in the relative abundance of bifidobacteria. In this study, we injected stools from responder and non-responder mice into colon organoids derived from a single young C57BL/6J animal to determine if, by using organoids derived from primary tissue from a single animal colonized with stools from multiple mice and treatments, we were capable of reproducing the bifidogenic response-no response effect observed in vivo. Stools from individual mice were processed, mixed with either GOS or PBS (control), and injected into organoids derived from the colon as previously described [73]. Organoids were collected immediately following injection, and at 24 and 72 h after injection for 16S rRNA amplicon sequencing and quantitative PCR analysis. Both, sequencing (Fig. 6a) and qPCR data (Fig. 6b) showed that the response (continuous lines in the figure) and non-response (represented as dashed lines) phenotypes were reproduced in the organoids injected with stools from both old and young mice, albeit the response was less pronounced in organoids injected with stools from old animals compared to young. Shannon diversity index and species richness values showed a rapid reduction soon after injection (Fig. 6c), with no statistically significant differences between treatments (GOS or PBS) or between young and old samples at any time (except at baseline) in contrast with the GOS diversity decrease observed in vivo. Principal coordinate analysis (PCoA) of samples showed compositional significant differences between the groups by age (PERMANOVA *P* value = 0.01) and time (*P* = 0.02), while the overall differences in microbiome composition between PBS and GOS-treated organoids approached significance (*P* = 0.06, not shown) (Fig. 6d).

At the compositional level, we observed clear differences between the original stool samples processed for injection, the microbiome of organoids injected with young versus old samples, and between the different treatments (GOS and PBS) (Supplementary Figures 3 and 4, and Supplementary Text). Our analysis allowed us to identify bacterial groups especially sensitive to manipulation, which were eliminated from the stool sample upon processing for injection into the organoids, potentially creating the niche for expansion of groups originally in very low numbers.

## Discussion

Our study confirmed previous reports indicating increased intestinal permeability (a “leaky gut”) during aging [19, 28]. Consistent with previously reported data [17], old mice had a distinct microbiome, increased ratios of non-saccharolytic versus saccharolytic bacteria, and correspondingly, lower abundance of β-galactosidases (EC:3.2.1.23), phospho-β-galactosidases (EC:3.2.1.85), and arabinogalactan endo-1,4- β-galactosidases (EC: 3.2.1.89). Based on numerous studies detailing the beneficial effects of prebiotic GOS, which include modulation of the gut microbiome with specific increases in beneficial bacteria [45, 47], stimulation of tight junctions and enhancement of the intestinal barrier function through modulation of goblet cells [38], reduction of inflammatory markers release in cell culture models [39], support of intestinal development and mucosal immune responses [40], and reduction in adherence of enteropathogenic *Escherichia coli* to tissue culture cells [74], we designed a short-term feeding trial to determine the effects of GOS on the aging gut microbiome and gut homeostasis. Our study also aimed to assess the transient effects of GOS on gut responses to antibiotic administration.

In contrast with a previous report [17], old animals had a significantly higher abundance of *Akkermansia*. *A. muciniphila* has been shown to reduce inflammation [75] and improve intestinal barrier integrity [76] suggesting that controlled mucolytic activity could benefit host physiology. However, uncontrolled degradation of mucins by proteolytic bacteria can increase inflammation, damage the barrier integrity, and increase susceptibility to pathogen infection [28, 77]. Old mice had a lower representation of *Bacteroides*. Although highly variable, the abundance of *Bacteroides* in aging human individuals is also lower than in the adult population [78]. GOS had a clear impact on the composition of the gut microbiome, increasing mostly the abundance of non-saccharolytic bacteria (*A. muciniphila*) but also saccharolytic species (genus *Bacteroides*, unknown species of the order *Bacteroidales*, and species of *Lactobacillus*). It has been previously reported that dietary GOS increased the abundance of bifidobacteria in human adults [41, 47] and older adults [55]. We also showed that GOS induced a variable bifidogenic response in 8–12-week-old mice [45]. In this study, we did not observe a consistent significant increase in the abundance of bifidobacteria in old or young animals fed the GOS diet. However, old mice did have a reduced proportion of bacterial β-galactosidases (EC:3.2.1.23), suggesting a reduction in metabolic potential of the microbiota. When fed GOS diet, both old and young animals showed an increased abundance of β-galactosidases. Reduced abundance of intestinal saccharolytic enzymes in the aging gut has been reported (although data is not conclusive) [79, 80] and may have important nutritional implications for the digestion of dietary carbohydrates by elderly individuals.

Administration of the GOS diet reduced bacterial diversity significantly in young and old mice. This is in accordance with the studies of GOS-supplemented infant formula [81] and in contrast with previous studies on human adults [47, 82] and young or adult BALB/c mice that showed no effect of GOS on bacterial diversity [83, 84]. Alpha diversity further decreased in young animals after administration of the antibiotic cocktail in the drinking water and remained stable throughout the remaining course of the study. The antibiotic treatment was selected based on the regime used in the murine model of *C. difficile* infection developed by Chen et al. [85] and includes an initial antibiotic cocktail in the drinking water that contains kanamycin, gentamicin, colistin, metronidazole, and vancomycin at a sufficient dose to disrupt the microbiome and increase the susceptibility to infection without major side effects. Reikvam et al. [86] reported that mice refrained from drinking metronidazole in water and lost weight under such treatment, possibly due to the concomitant reduction in food intake; however, in our study, animals did not lose weight during the antibiotic treatments. Likewise, none of the animals exhibited any signs of gastrointestinal distress or reduced activity before the clindamycin IP injection with temporary runny stools after the administration of the second antibiotic.

Clindamycin, depending on the organism, infection site, and concentration, can act as a bacteriostatic or bactericidal antibiotic. This antibiotic prevents the formation of peptide bonds effectively inhibiting protein synthesis by binding to the 50S ribosomal subunit. Clindamycin palmitate is hydrolyzed in the gastrointestinal tract and then distributed across the body [87]. This antibiotic has been associated with a high incidence of antibiotic-associated diarrhea [88]. In our study, the antibiotic caused an expected decreased abundance of *Bifidobacterium* in young and old mice on both diets. This is in agreement with a previous in vitro report that showed that, in contrast with the antibiotics tetracycline and ciprofloxacin, GOS feeding after clindamycin administration did not result in increased abundance or recovery of bifidobacteria populations [89].

Transcriptomics analysis of the colon at the end of the experiment indicated that GOS impacted intestinal expression differently in young compared to old mice. This differential modulation of host gene expression likely plays a critical role in the differences observed in host physiology, as well as in differential modulation of the microbiota composition. Old animals fed the GOS diet had increased expression of genes involved in small molecule metabolic processes (GO:0044281) and specifically the respirasome (GO:0070469). Although the role of these respiratory enzymes organized into supercomplexes in the intestine has not been defined, it could be hypothesized that they would reduce oxidative damage increasing metabolism efficiency [90, 91].

The effect of GOS on colonic gene expression of young mice showed a significant enrichment in binding-related genes (GO:0005488). Our study reports for the first time the specific in vivo induction of the galectin gene *Lgals1*, a β-galactosyl-binding lectin that bridge molecules by their sugar moieties, forming a signaling and adhesion network [69] by GOS diet. Previous studies showed that dietary supplementation with a mixture of short-chain GOS and long-chain fructo-oligosaccharides (lcFOS) in a 9:1 ratio could be involved in the maturation of the immune response in infants and induction of oral tolerance, hence reducing the risk of developing an allergic disease [92]. Likewise, this prebiotic mix suppressed the development of acute allergic symptoms, possibly by inducing regulatory T cells [93, 94]. The exact mechanisms of these effects are not known; however, a study suggested that prebiotics could modulate the immune response through galectins [95]. Intestinal epithelial cells are crucial in maintaining homeostasis and directing mucosal immune responses. They express Toll-like and Nod-like receptors that recognize antigens present in the intestinal lumen. When the Toll-like receptors are activated, intestinal epithelial cells contribute to the modulation of immune responses by secreting soluble mediators that bridge luminal signals with the immunological tissues. Both antigen-presenting cells and intestinal epithelial cells express proteins involved in the recognition of carbohydrate (glycan) structures present on bacterial and dietary components or glycosylated membrane proteins expressed by other cell types. Galectin-1 in particular have anti-inflammatory properties by enhancing the expansion and IL-10 production of regulatory T cells, resulting in the suppression of pro-inflammatory cytokines, including IFN-γ and TNF-α by effector T cells [96, 97]. Further analysis showed higher expression levels of genes in focal adhesion, PI3K-Akt, and ECM-receptor interaction pathways [71]. Cell-matrix adhesions play essential roles in cell motility, cell proliferation, cell differentiation, regulation of gene expression, and cell survival. At the cell-extracellular matrix contact points, specialized structures (focal adhesions), collections of actin filaments anchored to transmembrane receptors of the integrin family through a multi-molecular complex of junctional plaque proteins, act as structural links between membrane receptors and the actin cytoskeleton, or as signaling molecules, including different protein kinases and phosphatases, their substrates, and various adapter proteins [98, 99].

Microbiome research using colonic organoids as an in vitro platform is at its infancy [54, 100]. In this study, we took advantage of this platform to determine if the bifidogenic responder-non-responder phenotype [41, 45, 47] could be replicated in vitro. Injections of stools showed that the responder and non-responder phenotypes were reproduced in the organoids injected with stools from old and young mice. This allowed us to speculate that the primary contributor to the phenotype is the presence or absence of specific taxa and specific genetic components of the microbiota. Stool-injected organoids showed a rapid decrease in bacterial diversity after injection and showed compositional significant differences between the groups by age of the donor animal, time post-injection, and GOS treatment. This rapid decline in microbial diversity post-injection is likely due in part to oxygen exposure and limited nutrients; however, it may also be a consequence of the absence of host factors that facilitate microbial growth and colonization. In our study, the principal contributor to changes in the composition of the injected organoids was time. However, the differences in the microbiome associated with age were maintained over time, and minor differences attributable to the prebiotics were observed. This suggests that it could be possible to modify the experimental design to detect biologically relevant microbiome differences induced by treatment. The analysis of microbiome composition over time allowed us to identify bacterial groups especially sensitive to manipulation that were radically eliminated from the stool sample upon processing for injection into the organoids, creating a niche for expansion of groups originally in very low numbers in the stool samples. It could be argued that the differences in abundances of those bacterial groups help to explain the differences in the GOS bifidogenic response in vivo; however, the perturbation associated with the preparation of stool samples for injection into organoids was substantial causing the sharp decline in bacteria sensitive to such manipulations. Intestinal communities are unlikely to experience such disruptions naturally, and hence, it seems unlikely that the bacteria reduced by manipulation for organoid injection play a role in bifidogenic response in vivo.

The most remarkable effect of prebiotics in the old animals was the reduction of intestinal permeability and increased mucus biosynthesis. Defects in the intestinal barrier associated with aging have been previously reported [101, 102]. A relevant study by Thevaranjan et al. [28] reported that intestinal permeability increased with age due to age-associated microbial dysbiosis. In their study, increased permeability led to increased systemic inflammation with high levels of serum IL-6. In our study, we observed statistically non-significant increased IL-6 levels in the old control mice that were given antibiotics, but not in the non-antibiotics group. IL-6 were further increased in both young and old animals in the antibiotics-GOS groups. Increased serum IL-6 has been reported in neonate mice upon administration of antibiotics [103]. Although more studies are needed, we could speculate that the effect of GOS potentiated the impact of antibiotics, in both cases through modulation of the gut microbiome.

## Conclusions

Our study showed that dietary GOS modulated homeostasis of the aging gut by promoting changes in the microbiome composition and host gene expression, which was translated into decreased intestinal permeability and increased mucus production. It is not clear from this study if such modulation occurred by direct GOS-host interactions or exclusively via modulation of the microbiome. This study also demonstrated that age is a deciding factor on how prebiotics impact the microbiome and expression of intestinal epithelial cells. This was especially evident from the induction of the galectin-1 gene in young but not old mice, which has incredible implications on the modulation of the immune response. The present study did not attempt to correlate the transcriptome to differences in the microbiome; however, future studies will elucidate the complex interactions occurring during modulation of the host-microbes ecosystem by prebiotics.

## Methods

### Animal housing, treatment, and sample collection

Forty-eight female C57BL/6J SPF mice (24 6-week-old and 24 60-week-old) received a control diet (D17121301; Research Diets INC.) for a 2-week co-housed (6/cage) normalization period. Animals were subsequently paired off (1 old/1 young) from different normalization cages and separated into groups fed control or GOS diets, using an optimized GOS diet (D17121302; Research Diets INC.) replacing 71.8 g of cellulose with 71.8 g of pure GOS per kilogram. Pure prebiotics were generated by heterologous expression of the beta-hexosyl transferase from *Sporobolomyces singularis* in *Pichia pastoris* as previously described [45, 49]. After 2 weeks, half of the animals in each group (6 young and 6 old from each diet) were administered an antibiotic cocktail in their drinking water for 3 days according to the *C. difficile* infection protocol described by Chen et al. [85]. The cocktail contained kanamycin (40 mg/kg), gentamycin (3.5 mg/kg), colistin (4.2 mg/kg), metronidazole (21.5 mg/kg), and vancomycin (4.5 mg/kg). The concentrations of antibiotics in water were calculated based on the average weight and expected water consumption of mice. After 3 days, the water was replaced with antibiotic-free water, and the animals were allowed 2 days to recover prior to receiving an intraperitoneal injection of clindamycin (10 μg/g body weight). Animals remained on their respective diets and were sacrificed following a 1-week recovery period. For both experiments, fresh stool samples were collected and stored at − 80 °C. Intestinal tissues and contents were collected at termination (Fig. 2a). Animal weight and behavior were monitored throughout the study.

### Mouse intestinal permeability assay

Mice were administered 100 mg fluorescein isothiocyanate (FITC) dextran/100 g body weight via oral gavage 4 h prior to sacrifice. Immediately following euthanasia, blood was harvested via cardiac puncture, and the serum was subsequently separated via centrifugation. The serum from each animal was assayed for the presence and quantity of FITC signal with a TECAN Infinite M200 plate reader, using an excitation wavelength of 485 nm and an emission wavelength of 528 nm. A standard curve of FITC dextran was used to quantify the signal in each serum sample.

### Mucin staining and tissue imaging

The sections of mouse distal colon were harvested, embedded in optimal cutting temperature (OCT) compounding agent, and flash-frozen in a dry ice-filled ethanol bath without fixation. Blocks were stored at − 80 °C until cut at − 20 °C on a cryostat, and frozen sections were mounted onto slides. The sections were immediately subject to paraformaldehyde vapor fixation (4% PFA, 60C) for 8 h prior to PAS staining and subsequent imaging on Nikon 2000-E inverted widefield microscope. Images were saved as RFG files and uploaded to the ImageJ Analysis software, where pixel analysis was performed to quantify the abundance of mucin/mucin-producing cells (blue/purple) and epithelial cells (pink).

### Organoid cultivation and colonization

Organoids were derived from colon crypts harvested from a single young C57BL/6J and grown in 96-well plates embedded in Corning® Matrigel® matrix (Corning Inc., Corning NY) and overlaid with complex DMEM-F12 growth media containing 1 μg/ml Pen Strep [104, 105]. Organoids were incubated at 37 °C under 5% CO_2_ and ambient oxygen and were passaged into new plates every 10–14 days. Organoids used for injection were grown 4 days post-passage and were uniform in size and shape. Homogenized stool samples collected from both young and old donor mice prior to GOS treatment were filtered through 5-μM syringe filters (Millipore) and loaded into microinjection needles for organoid inoculation as previously described [54]. Loaded needles were attached to the injection apparatus, and organoids were injected with ~ 400 pL of stool suspension. Injected organoids were then incubated at 37 °C under 5% CO_2_ and ambient oxygen with supplemental antibiotics. Organoids were harvested at 0 h, 24 h, and 72 h post-injection.

### Nucleic acid isolation

DNA from fecal samples and microbiota-colonized organoids were extracted using the Qiagen DNeasy stool DNA isolation kit (Qiagen, Valencia, CA) with an additional bead-beating step aimed to ensure uniform lysis of bacterial spores. Samples were loaded into tubes containing 10 mg of sterile, acid washed, 1 μm glass beads, and homogenized for 5 min at 15 Hz in Qiagen TissueLyser II (Qiagen, Valencia, CA). DNA was subsequently used for 16S rRNA amplicon sequencing and whole-genome shotgun sequencing. RNA isolation from tissues was performed using the Qiagen RNeasy kit (Qiagen, Valencia, CA) following the manufacturer’s instructions for subsequent use in RTqPCR and mRNA sequencing.

### High-throughput quantitative PCR detection of *Bifidobacterium*

The Access array AA 24.192 (Fluidigm Corporation, San Francisco, CA) was used in the UNC Advanced Analytics Core. Primers for amplification of the 16S rRNA gene and GroEL have been validated in previous studies [106–109]. The taxonomic groups targeted in the *Bifidobacterium* array include domain *Bacteria*, phylum *Actinobacteria*, genus *Bifidobacterium*, and *Bifidobacterium* species. Pre-amplification (specific target amplification (STA)) assays and microfluidic qPCR were be performed on a BioMark HD reader as described [45]. Raw data were normalized using the Livak method [110]. Cq values for each sample were normalized against their respective Cq value obtained from universal primers using the equation: ratio (reference/target) = 2^−Ct (ref)-Ct (target)^.

### 16S rRNA amplicon sequencing

Total DNA was subject to amplification of the V4 region of the 16S rRNA gene using primers (515F-806R) with Illumina adaptors. Amplicons were barcoded using Illumina dual-index barcodes (Index 1(i7) and Index 2(i5)), purified using Agencourt® AMPure® XP Reagent (Beckman Coulter, Brea, CA) and quantified with Quant-iT™ PicoGreen® dsDNA Reagent (Molecular Probes, Thermo Fisher Scientific, Waltham, MA). Libraries were pooled in equimolar amounts and sequenced on MiSeq (Illumina, San Diego, CA).

#### Data analysis

Sequencing output from the Illumina MiSeq platform was converted to fastq format and demultiplexed using Illumina Bcl2Fastq 2.18.0.12. The resulting paired-end reads were processed using QIIME 2 [111] 2018.11. Index and linker primer sequences were trimmed using the QIIME 2 invocation of cutadapt. The resulting paired-end reads were processed with DADA2 through QIIME 2 including merging paired ends, quality filtering, error correction, and chimera detection. An average of 81,904 filtered, denoised, merged, non-chimeric sequences wase produced per sample. Amplicon sequencing units from DADA2 were assigned taxonomic identifiers using Green Genes release 13_08. Alpha diversity indexes (Faith PD whole tree, EVENNESS (Shannon), and observed species) were estimated using QIIME 2 at a rarefaction depth of 10,000 sequences per sample. Beta diversity estimates were calculated within QIIME 2 using weighted and unweighted UniFrac distances as well as Bray-Curtis dissimilarity between samples at an initial subsampling depth of 5000 and then 1000 since antibiotic-treated samples had lower yields than non-antibiotic samples. The results were summarized and visualized through principal coordinate analysis, and significance was estimated as implemented in QIIME 2. Significance of differential abundance was estimated using ANCOM as implemented in QIIME 2.

### Assignment of saccharolytic (SAC) and non-saccharolytic (NON_SAC) to bacterial taxa

We used the reference study by Vieira-Silva et al. [112], which mined 532 publicly available gut reference genomes and assigned them to four different groups (proteolytic, saccharolytic, lipolytic, and generalist bacteria) using metagenome analytical methods, and the study by Magnusdottir et al. [113] on the metabolic reconstruction network AGORA. In the case that the genus was not categorized in the mentioned studies, we referred to the *Bergey’s Manual of Systematic Bacteriology* [114] and previously published reports when the genus was not found in either source [115–126]. We included saccharolytic and generalist bacteria in the SAC group, as well as chemolithoheterotrophic bacteria capable of using either carbohydrates or the metabolites derived from carbohydrate sources (for example, *Dehalobacter*, *Geobacillus*). Likewise, the NON_SAC category included proteolytic and lipolytic bacteria.

### Whole-Genome Shotgun (WGS) sequencing

One nanogram of intact genomic DNA was processed using the Nextera XT DNA Sample Preparation Kit (Illumina, San Diego, CA). In this process, the target DNA was simultaneously fragmented and tagged by the Nextera Enzyme Mix containing transposome, which fragments the input DNA and adds bridge PCR (bPCR)-compatible adaptors required for binding and clustering on the flow cell. Next, fragmented DNA was amplified via a limited-cycle PCR program adding index 1(i7) and index 2(i5) (Illumina) in a unique combination for each sample, as well as primer sequences required for cluster formation. Libraries were purified using Agencourt® AMPure® XP Reagent (Beckman Coulter, Brea, CA) and quantified with Quant-iT™ PicoGreen® dsDNA Reagent (Molecular Probes, Thermo Fisher Scientific, Waltham, MA). All libraries were pooled in equimolar amounts and heat-denatured before loading on Illumina HiSeq2000 Rapid.

#### Data analysis

Sequencing output from the Illumina platform was converted to fastq format and demultiplexed using Illumina Bcl2Fastq 2.18.0.12. An average of 11 million reads was generated per sample. Quality control of the demultiplexed sequencing reads was verified by FastQC. The resulting paired-end reads were aligned with Bowtie2 [127] against the host reference, and all aligning reads will be eliminated. Paired-end reads were joined with vsearch 1.10.2. The resulting single-end reads were again aligned against the reference with Bowtie2 retaining all reads that did not align. Estimates of taxonomic composition, gene family, path abundance, and path coverage were produced from the remaining reads using HUMAnN2 [128].

### Reverse transcription qPCR

1.5 μg of total RNA was subject to reverse transcription using the iScript Advanced cDNA synthesis kit (Bio-Rad, Hercules, CA). Total nucleic acid was subsequently quantified and normalized to 1 ng/μl prior to qPCR setup. qPCR reactions were performed using the PowerSYBR Green Master Mix, 3 ng of total nucleic acid per reaction, and the following primer pairs (at a final concentration of 100 nM): TNFa (Fwd: 5′-ACGGCATGGATCTCAAAGAC-3′, Rev: 5′-GTGGGTGAGGAGCACGTAGT-3′) [129], IL-6 (Fwd: 5′-CTGCAAGAGACTTCCATCCAGTT-3′, Rev: 5′-GAAGTAGGGAAGGCCGTGG-3′) [130], GapDH (Fwd: 5′-TGCACCACCACCAACTGCTTAG-3′, Rev: 5′-GGATGCAGGGATGATGTTC-3′) [131], Muc2 (Fwd: 5′-GCTGACGAGTGGTTGGTGAATG-3′, Rev: 5′-GATGAGGTGGCAGACAGGAGAC-3′) [132], RELMβ (Fwd: 5′-CCATTTCCTGAGCTTTCTGG-3′, Rev: 5′-AGCACATCCAGTGACAACCA-3′) [133], and TFF3 (Fwd: 5′-CAGATTACGTTGGCCTGTCTCC-3′, Rev: 5′-ATGCTTGCTACCCTTGGACCAC-3′) [133]. qPCR reactions were performed on the QuantStudio Q6 instrument (Thermo Fisher Scientific, Waltham, MA). The samples were run in technical triplicate, and included no template, and no reverse transcriptase controls in addition to the internal GapDH control for normalization of gene expression. Cycle threshold values (CT) were calculated from amplification plot data by the QuantStudio6 software at the completion of each qPCR run. CT values were normalized to the internal GapDH controls to generate ΔCT values for each gene in each animal. ΔΔCT values were generated by comparing the ΔCT values from control animals within an age group to the ΔCT values from experimental animals within that same group. Fold change (FC) of gene expression of each target gene between groups was calculated by using the following equation: FC = 2^(−ΔΔCT).

### Mouse mRNA sequencing

Total RNA isolated from mouse colon was processed using the NuGEN Universal Plus mRNA-Seq kit (NuGEN Technologies, Inc., San Carlos, CA) for whole transcriptome sequencing as directed by the manufacturer. Briefly, total RNA was subject to poly(A) selection, fragmentation, first-strand synthesis, second-strand synthesis, end repair, adaptor ligation, strand selection, and finally library amplification. Indexed cDNA libraries quantified via Quant-iT™ PicoGreen® dsDNA reagent (Thermo Fisher Scientific, Waltham, MA) were pooled at equimolar concentrations and sequenced on Illumina HiSeq4000 platform.

#### Data analysis

Demultiplexed paired-end reads from mRNA sequencing experiments were aligned with STAR [134] against the mouse Mm9 reference. Salmon [135] was applied to the resulting alignment to estimate the quantity of transcript expression. The significance of differential expression was measured with DESeq2 [136].

### Cytokine analysis

Serum was subject to Milliplex cytokine/chemokine assay MCYTOMAG-70K (Millipore, Sigma, Burlington, MA) for the detection and quantification of TNFα, IP-10, IL-17, IL-13, IL-10, IL-6, IL-4, IL-1α, eotaxin, IL-12, and IL-7. The assay was run at the UNC Advanced Analytics Core on DropArray™96 Plate system (Curiox Biosystems, San Carlos, CA) as recommended by the manufacturer.

### Statistical analyses

Group differences were tested for statistical significance using ANOVA with ad hoc Tukey tests for pairwise comparisons, and *P* values were reported accordingly. Differences in the microbiota composition were determined using non-parametric tests (Kruskal-Wallis or Mann-Whitney as appropriate) and analysis of composition of microbiomes (ANCOM) [137] analyses. FDR correction was applied to the statistical analysis of samples to take into consideration multiple comparisons. Differences in beta diversity were determined using analysis of similarity (ANOSIM) and permutational multivariate analysis of variance (PERMANOVA) analyses in QIIME2. Statistical differences in the relative abundance of beta-galactosidases were determined using the Kruskal-Wallis *H* test with FDR correction in STAMP [138]. Unless otherwise indicated, the cutoff for statistical significance was set to *P* < 0.05 for the determination of differences between the groups in the microbiota, gene expression, intestinal permeability, and cytokine analyses.

## Supplementary Information

**Additional file 1: Figure S1.** Relative abundance of *Bacteroides* (a), *Akkermansia muciniphila* (b), and *Lactobacillus* (c) were increased by GOS diets. Abundance of *Clostridium* (d), *Adlecreutzia* (e) and *Ruminococcus* (f) were reduced by GOS diets.

**Additional file 2: Figure S2.** Individual pixel-pigment analysis of mucus staining of tissue samples revealed a distinctly higher abundance of pixels in the lower range of the pigment spectrum (darker colors, blue and purple) in GOS-fed old animals, compared to young or control-fed old animals (a). Pigment-specific histograms of the RGB image files were generated using ImageJ software, revealing differences between samples in the abundance of pixels in each image, as well as providing quantification of pixels within each pigment range (b). Ratios between epithelial and mucosal pixels were calculated and used in determining the fold change in pigment between specific pairs of animals, Old animals GOS vs Control diets, Young animals GOS vs Control diets, Old animals vs Young animals feeding on control diet, and finally Old animals vs Young animals feeding on GOS diet (c). Mucus fold change was calculated for each animal compared to Young animals fed control diet (d).

**Additional file 3: Figure S3.** Heatmap represents changes in bacterial abundance within old microbiota-colonized organoids over time when supplemented with either PBS (control), GOS, or Lactose.

**Additional file 4: Figure S4.** Heatmap represents changes in bacterial abundance within young microbiota-colonized organoids over time when supplemented with either PBS (control), GOS, or Lactose.

**Additional file 5.** Supplementary text.

**Additional file 6: Table S1.** Diet composition as provided by the manufacturers.

**Additional file 7: Table S2. ** Expression data from colons of young and old mice fed either the control or GOS diets.

## Data Availability

All sequencing data has been submitted to the NCBI repository and can be accessed via the following accession numbers: mouse microbiota 16S rRNA amplicon sequencing PRJNA605460, whole-genome shotgun sequencing PRJNA605640, mouse mRNA sequencing PRJNA605019, and organoid 16S rRNA amplicon sequencing PRJNA606062.
