# Correction to: The pleiotropic effects of prebiotic galacto-oligosaccharides on the aging gut

**DOI:** 10.1186/s40168-021-01030-z

**Published:** 2021-02-26

**Authors:** Jason W. Arnold, Jeffery Roach, Salvador Fabela, Emily Moorfield, Shengli Ding, Eric Blue, Suzanne Dagher, Scott Magness, Rita Tamayo, Jose M. Bruno-Barcena, M. Andrea Azcarate-Peril

**Affiliations:** 1 Department of Medicine, Division of Gastroenterology and Hepatology, School of Medicine, University of North Carolina, Chapel Hill, NC USA; 2 UNC Microbiome Core, Center for Gastrointestinal Biology and Disease (CGIBD), School of Medicine, University of North Carolina, Chapel Hill, NC USA; 3 UNC Information Technology Services and Research Computing, University of North Carolina, Chapel Hill, NC USA; 4 Current affiliation: Programa de Inmunología Molecular Microbiana. Departamento de Microbiología y Parasitología, Facultad de Medicina, Universidad Nacional Autónoma de Mexico, Mexico City, Mexico; 5 Department of Cell Biology and Physiology, University of North Carolina, Chapel Hill, NC USA; 6 Department of Plant and Microbial Biology, North Carolina State University, Raleigh, NC USA; 7 Joint Department of Biomedical Engineering, University of North Carolina, Chapel Hill and North Carolina State University, Raleigh, NC USA; 8 Department of Microbiology and Immunology, University of North Carolina, Chapel Hill, NC USA

**Correction to: Microbiome 9, 31 (2021)**

**https://doi.org/10.1186/s40168-020-00980-0**

Following publication of the original article [1], an error was identified in Fig. 4. The correct figure is given below.

**Fig. 4:**
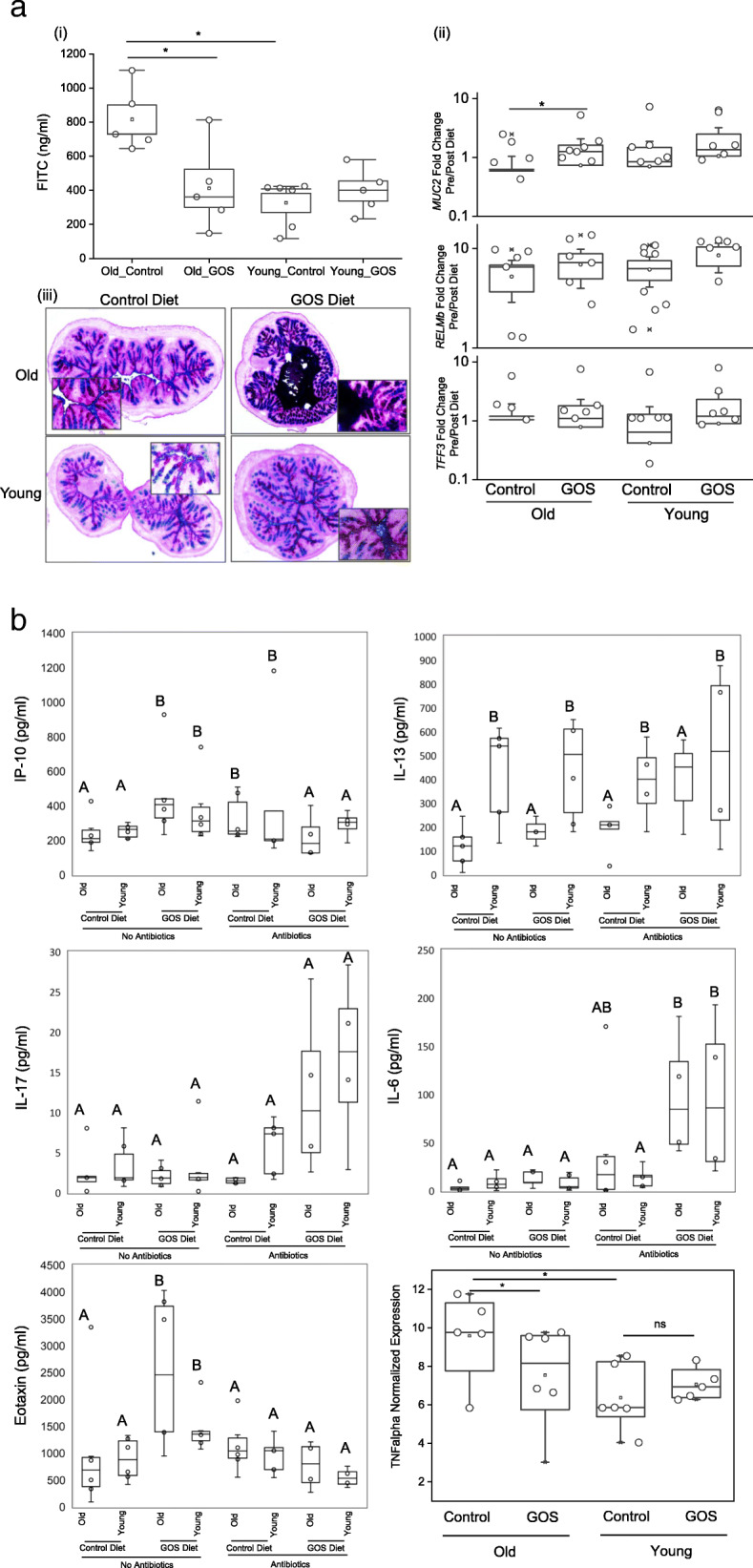
**a** (i) Old mice had higher intestinal permeability measured by FITC-dextran assays than young animals. (ii) Old mice fed GOS had significantly increased *MUC2* expression (*p < 0.05). The expression of *TFF3* and *RELMb* tended to increase in the GOS groups, but differences were not statistically significant. (iii) Paraformaldehyde vapor fixation and subsequent PAS staining showed increased mucus thickness in old mice fed the prebiotics diet. **b** Inflammatory biomarkers were modulated by antibiotics and GOS. A 2 × 2 × 2 ANOVA test showed (i) increased serum IP-10 in GOS-fed animals without antibiotic treatment and in antibiotic-treated animals fed control diet. (ii) Serum IL-13 levels were higher in young animals than in old animals in all groups. (iii) IL-17 levels were higher in antibiotic-treated animals than in animals without antibiotics. (iv) IL-6 was increased in antibiotic-treated old animals (GOS and control) compared to old animals without antibiotic treatments and elevated in young animals treated with both GOS and antibiotics. (v) Eotaxin levels were higher in GOS-fed animals without antibiotic treatment, but lower in GOS-fed animals that received antibiotics, regardless of age. (vi) Expression of TNFα quantified by RT-qPCR was higher in old animals compared to young and reduced by GOS treatment in old animals
