# Supplementary material for: The pleiotropic effects of prebiotic galacto-oligosaccharides on the aging gut
Source: Microbiome. 2021 Jan 28;9:31. doi: 10.1186/s40168-020-00980-0 (PMC7845053; doi:10.1186/s40168-020-00980-0)
Supplement: Supplementary file 2 — Additional file 1: Figure S1. Relative abundance of Bacteroides (a), Akkermansia muciniphila (b), and Lactobacillus (c) were increased by GOS diets. Abundance of Clostridium (d), Adlecreutzia (e) and Ruminococcus (f) were reduced by GOS diets. [file 40168_2020_980_MOESM1_ESM.docx]

A

*

*

B

*

*

D

*

*

F

*

C

**

*

E

**

**

*

p<0.01

**

p<0.1

Supplementary Figure 1
