# Supplementary figures and images for: The pleiotropic effects of prebiotic galacto-oligosaccharides on the aging gut
Source: Microbiome. 2021 Jan 28;9:31. doi: 10.1186/s40168-020-00980-0 (PMC7845053; doi:10.1186/s40168-020-00980-0)

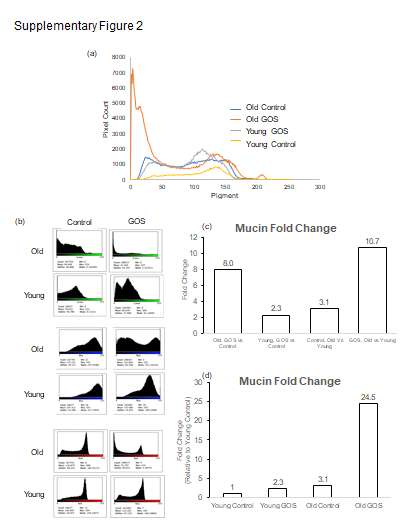

Supplement: Supplementary file 3 — Additional file 2: Figure S2. Individual pixel-pigment analysis of mucus staining of tissue samples revealed a distinctly higher abundance of pixels in the lower range of the pigment spectrum (darker colors, blue and purple) in GOS-fed old animals, compared to young or control-fed old animals (a). Pigment-specific histograms of the RGB image files were generated using ImageJ software, revealing differences between samples in the abundance of pixels in each image, as well as providing quantification of pixels within each pigment range (b). Ratios between epithelial and mucosal pixels were calculated and used in determining the fold change in pigment between specific pairs of animals, Old animals GOS vs Control diets, Young animals GOS vs Control diets, Old animals vs Young animals feeding on control diet, and finally Old animals vs Young animals feeding on GOS diet (c). Mucus fold change was calculated for each animal compared to Young animals fed control diet (d). [file 40168_2020_980_MOESM2_ESM.docx]

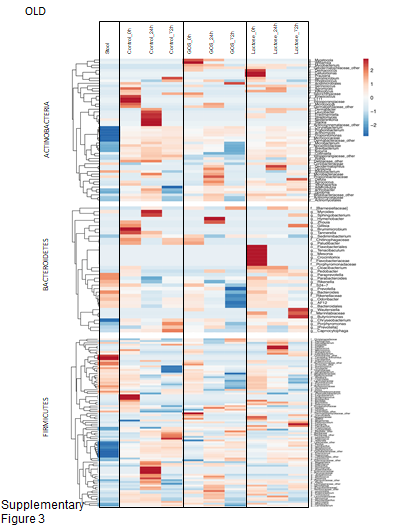


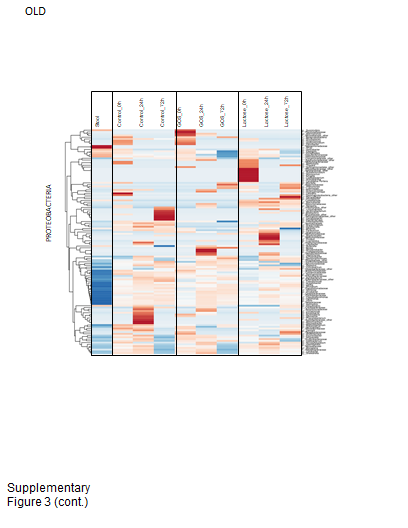

Supplement: Supplementary file 4 — Additional file 3: Figure S3. Heatmap represents changes in bacterial abundance within old microbiota-colonized organoids over time when supplemented with either PBS (control), GOS, or Lactose. [file 40168_2020_980_MOESM3_ESM.docx]

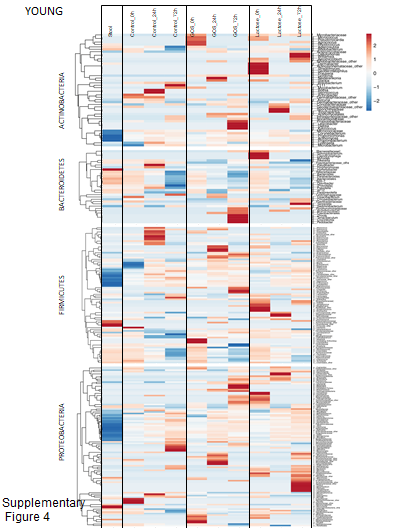

Supplement: Supplementary file 5 — Additional file 4: Figure S4. Heatmap represents changes in bacterial abundance within young microbiota-colonized organoids over time when supplemented with either PBS (control), GOS, or Lactose. [file 40168_2020_980_MOESM4_ESM.docx]
