# Supplementary material for: The pleiotropic effects of prebiotic galacto-oligosaccharides on the aging gut
Source: Microbiome. 2021 Jan 28;9:31. doi: 10.1186/s40168-020-00980-0 (PMC7845053; doi:10.1186/s40168-020-00980-0)
Supplement: Supplementary file 6 — Additional file 5. Supplementary text. [file 40168_2020_980_MOESM5_ESM.docx]

**Supplementary Text**

**Clindamycin impact on the gut microbiome.** Analysis of fold change differences between groups that received or not an IP injection of clindamycin showed that both age and diet impacted compositional changes. The antibiotic caused significant decreases (FDR corrected *P*<0.05) *Enterobacteriaceae_other, Clostridiaceae_other, Turicibacter, Streptococcus, Lactococcus, Peptostreptococcaceae, Allobaculum,* *Enterococcus*, and *Bifidobacterium* in both young and old mice fed the control diet, while *Bacillaceae_other, Propionibacterium, Lactobacillus,* and *S24-7* decreased only in old controls and *RF39* decreased only in young animals. Conversely, clindamycin resulted in increased abundance of *Clostridiales, Clostridium, Paenibacillus, [Mogibacteriaceae], Ruminococcaceae_other, Anaerotruncus, Ruminococcus, Epulopiscium, Erysipelotrichaceae, Coprococcus, Dorea, Lachnospiraceae_other, Oscillospira, Akkermansia, Ruminococcaceae, Bacteroides, [Ruminococcus], Sutterella,* and *Bacillus* in both young and old mice in the control groups while *S24-7* increased only in young and *Adlercreutzia* and *Rikenellaceae* increased only in old animals.

A comparative analysis between the antibiotic groups in the GOS diets revealed that in both young and old mice, the abundance of *Bacteroides, Bifidobacterium, Enterobacteriaceae_other, Lactococcus,* *S24-7* and *Allobaculum* decreased in both old and young GOS-antibiotic groups while *Lachnospiraceae, Erysipelotrichaceae,* and *Turicibacter* decreased only in old mice and [*Mogibacteriaceae*] decreased only in young. Conversely, *Clostridium, Clostridiales_other, Ruminococcus, Bacillaceae_other, Enterococcus, Akkermansia*

*Coprococcus* and *Sutterella* increased in both old and young GOS-fed mice, while *Bacillus* and *Staphylococcus* increased only in old, and *Oscillospira*, and *Paenibacillus* increased significantly in young mice only.

**GOS impact on the microbiome of colonic organoids injected with stools from old and young mice.** Analysis of the microbiome of filtered stools and organoids injected with stool plus PBS (control), GOS or lactose at the species level for the phyla *Actinobacteria*, *Bacteroidetes*, *Firmicutes* and *Proteobacteria* showed clear differences between the original stool samples and also between the different groups indicating that the stool microbiota is active at collection and changes in composition occur rapidly after.

In organoids injected with stools from old animals, within the Actinobacteria, we observed increased *Corynebacterium, Propionibacterium, Actinomyces, Propionicymonas, Micrococcaceae*, and *Dermabacteraceae_other* across all samples and treatments suggesting either a low baseline level followed by a bloom induced by environmental conditions or an environmental contamination. The control group at time 0h had an increased abundance of rare taxa including *Kineococcus,* C111, Intrasporangiaceae, *Micrococcocus*, and *Dermatophilaceae_other*. These bacteria were reduced in subsequent time points. This was followed by a bloom at 24 hours of *Dermabacter, Leucobacter, Friedmanniella, Streptomyces, Nesterenkonia, Slackia* and Actinosynnemataceae_other. The GOS group at time 0h had increased *Mycetocola, Williamsia, Mycobacterium*, *Geodermatophilaceae*_*other*, and *Dermacoccus*. This was followed at 24h by a bloom in *Geodermatophilus, Oerskovia, Bifidobacterium, Microbacteriaceae, Propionibacteriaceae, Dietzia, Agrococcus*, and *Yonghaparkia*, which decreased after prebiotic depletion, along with the taxa *Microbacterium*, Nocardioidaceae, *Brevibacterium, Kocuria* and *Collinsella*. The lactose group started with high abundance of *Dermacoccus, Cellulomonas*, and *Prauseria*, which decreased at subsequent time points.

In the *Bacteroidetes* phylum, we observed variable changes in the abundance of *Chryseobacterium, Porphyromonas, [Prevotella*], and *Capnocytophaga* across treatments. This cluster was increased at 72 hours in the control and lactose groups but decreased in the GOS group at the same time point. Conversely, the cluster including the taxa *Paraprevotella, Parabacteroides, Rikenella, S24-7, Prevotella, Bacteroides*, and *Odoribacter*, which had a high abundance in the stool sample, showed a decreased abundance over time in all groups, specially at 72 hours in the GOS group. The GOS organoids at 24 hours had high levels of *Hymenobacter* and *Zhouia*, and relatively increased abundance of *Chryseobacterium*, *Porphyromonas*, and [*Prevotella*], while the control had high abundance of *Myroides* and *Sphingobacterium* and the lactose group had relatively high levels of *Cloacibacterium*, *Pedobacter* and *Paraprevotella* at the same time point. The most drastic changes were observed in the lactose group which had, at 0 hours, very high levels of *Flavobacteriales*, *Tenacibacterium, Mesonia, Crocinitomix*, *Flavobacteriaceae*, and *Porphyromonadaceae* and at 72 hours high abundance of *Wautersiella*, Marinilabiaceae, and *Butyricimonas*.

The most represented phyla in organoids injected with stools from old animals was the *Firmicutes*. Our analysis revealed a number of taxa highly abundant in stools that were practically absent across groups (except at 72 hours in the lactose group): *Clostridiaceae_other*, Candidatus Arthromitus, and *Coprobacillus*. Conversely, a cluster of 9 taxa (*Listeriaceae_other, Bacillus, Lysinibacillus, Streptococcus, Staphylococcus, Carnobacteriaceae_other*, *Enterococcus, Vagococcus*, and *Lactobacillales_other)* were absent in stools but showed a variable increase over time across all groups. The GOS group at 24 hours had increased *Dialister, Leuconostoc*, and *Alloiococcus*, while at 72 hours we saw increases in *Christensenellaceae*, *Peptoniphilus,* *Bacillales_other*, *Lactococcus* and *Enterococcaceae_other.* Interestingly, at 24 hours, the lactose group showed marked increases in *Faecalibacterium, Finegoldia, Filifactor, Peptococcus, Macrococcus*, [Eubacterium], and *Catenibacterium.*

Finally, the phylum Proteobacteria showed similar patterns in terms of taxa present in stools but absent across samples (*Nitrosomonadaceae* and *RF32*) and taxa of reduced presence in stools with increased abundance in the organoids groups (Bradyrhizobiaceae, Comamonadaceae, *Sphingomonas*, Enterobacteriaceae, *Pseudomonas, Agrobacterium, Acinetobacter, Delftia, Nitrincola, Arcobacter,* *Oxalobacteraceae*, *Phyllobacteriaceae*, *Pseudoalteromonas, Halomonas, Vibrio*, Neisseriaceae, and *Salinivibrio*). The groups *Bosea, Rhodanobacter, Thalassomonas*, *Desulfohalobiaceae*, *Lepidimonas,* and *Enhydrobacter* were increased in the GOS group at 24 hours, while *Betaproteobacteria*, *Moritella*, Beijerinckiaceae, and *Alteromonas* increased at 72 hours.

Organoids injected with stools from young animals showed similar patterns of change in microbiome composition. Within Actinobacteria, the same cluster of taxa as in organoids injected with stools from old mice (*Propionibacterium, Actinomyces, Propionicimonas, Corynebacterium*, and *Micrococcaceae*) was increased across treatments. The GOS group at baseline had high levels of *Microbacteriaceae*, *Micrococcus, Pseudonocardia, Agrococcus*, and *Rhodococcus.* At 24 hours we observed increased abundance of *Brevibacterium* and *Slackia* (which decreased at 72 hours) and *Acinomycetales* (which persisted at 72 hours). At 72 hours, the GOS group had a high abundance of *Propionibacteriaceae*, *Leucobacter, Dietzia*, *Kytococcus*, and *Micrococcaceae*.

The organoids injected with stools from young mice (and the original pooled stool sample) had an overall lower diversity within the *Bacteroidetes* phylum, compared to stools from old animals. The genus *Hymenobacter*, highly represented in stools, decreased rapidly across all groups. As in the organoids injected with stools from old mice, the taxa including *Rikenellaceae*, *Bacteroides, Parabacteroides, S24-7,* and *Odoribacter* showed a decreased abundance over time in all groups. Conversely, the cluster comprising *Pedobacter, Crocinitomix, Tenacibaculum, Zhouia*, Flavobacteriales, *Brumimicrobium*, and Porphyromonadaceae, with low representation in the stool sample, showed a clear increase in the GOS group at 72 hours.

Within the Firmicutes, the cluster containing uncharacterized bacteria (*Peptostreptococcaceae*, *Ruminococcaceae*_*other*, *Clostridiaceae*_*other*, and *Firmicutes*_*other*), which was highly represented in stool, decreased across groups with the exception of GOS at 24 hours (increased *Ruminococcaceae*_*other*) and lactose at 72 hours (increased *Peptostreptococcaceae*). As in organoids injected with stools from old animals, a cluster with low representation in stools showed mostly increased abundance across groups. The cluster included *Enterococcaceae*_*other*, *Bacillus*, *Lactobacillales*_*other*, *Carnobacteriaceae*_*other*, *Streptococcaceae*, *Listeriaceae*_*other*, *Enterococcus, Vagococcus, Staphylococcus, Lysinibacillus, Planococcaceae*, and *Finegoldia*. The GOS group had an early bloom of Candidatus Arthromitus, *Coprobacillus*, and *Desemzia*, increased abundance at 24 hours of *Macrococcus, Peptococcus, Filifactor* and *Bacillales*_*other*, and increased abundance at 72 hours of *Planococcaceae, Finegoldia, Aerococcus,* and *Anaerobacillus*. GOS treatment also resulted in a marked reduction at 72 hours of *Roseburia* and *Clostridiaceae*.

Finally, within the phylum *Proteobacteria*, we observed a drastic reduction of *Marinobacter* and *Gammaproteobacteria*_*other* across treatments as well as increases in a large cluster that included *Rhizobiales*, *Pseudomonas, Vibrio*, *Neisseraceae*, *Enterobacteriaceae*, and *Delftia*. The GOS group had high levels of *Methylophilaceae*, *Kingella*, *Nitrosomonadaceae*, *Desulfohalobiaceae*, *Rhodospirillaceae*, and *Janthinobacterium* at baseline, increased *Hyphomicrobiaceae*, *Amaricoccus*, Methylobacteriaceae, *Rhodobacteraceae*_*other*, *Comamonadaceae*_other, *Moraxellaceae*_*other*, *Piscirickettsiaceae*, and *Betaproteobacteria* at 24 hours, and increased *Methylophilus, Bdellvibrio*, and *Candidatus* *Portiera* at 72 hours. This analysis allowed us to identify bacterial groups especially sensitive to manipulation, which could be drastically eliminated from the stool sample upon processing for injection into the organoids, creating the niche for expansion of groups originally in very low numbers.
