# Supplementary material for: The pleiotropic effects of prebiotic galacto-oligosaccharides on the aging gut
Source: Microbiome. 2021 Jan 28;9:31. doi: 10.1186/s40168-020-00980-0 (PMC7845053; doi:10.1186/s40168-020-00980-0)
Supplement: Supplementary file 7 — Additional file 6: Table S1. Diet composition as provided by the manufacturers. [file 40168_2020_980_MOESM6_ESM.docx]

**Table S1.** Diet composition as provided by the manufacturers.

|  | D17121301 (Control) | D17121302 (GOS) |
| --- | --- | --- |
| Ingredient (g/kg) |  |  |
| Casein | 200 | 200 |
| L-Cystine | 3 | 3 |
| Corn Starch | 433.2 | 413.5 |
| Maltodextrin | 110 | 110 |
| Dextrose/Sucrose | 100 | 100 |
| Lactose | 7.2 | 0 |
|  |  |  |
| Cellulose | 71.8 | 0 |
| GOS | 0 | 71.8 |
| Soybean Oil | 70 | 70 |
| Mineral Mix | 10 | 10 |
| Vitamin Mix | 10 | 10 |
| Dicalcium phosphate | 13 | 13 |
| Calcium carbonate | 5.5 | 5.5 |
| Potassium Citrate | 16.5 | 16.5 |
| Choline Bitartrate | 2 | 2 |
| TBHQ | 0 | 0 |
|  |  |  |
| Yellow Dye | 0 | 0 |
| Red Dye | 0 | 0.05 |
| Blue Dye | 0.05 | 0 |

D17121301 (Control) Research Diets Inc.

D17121302 (GOS) Research Diets Inc.
